# Virtual Rehabilitation for Multiple Sclerosis Using a Kinect-Based System: Randomized Controlled Trial

**DOI:** 10.2196/games.2933

**Published:** 2014-11-12

**Authors:** Jose-Antonio Lozano-Quilis, Hermenegildo Gil-Gómez, Jose-Antonio Gil-Gómez, Sergio Albiol-Pérez, Guillermo Palacios-Navarro, Habib M Fardoun, Abdulfattah S Mashat

**Affiliations:** ^1^Instituto de Automática e Informática IndustrialUniversitat Politècnica de ValènciaValenciaSpain; ^2^Departamento de Informática e Ingeniería de SistemasUniversidad de ZaragozaTeruelSpain; ^3^Information Systems Department King AbdulazizUniversity (KAU) JeddahJeddahSaudi Arabia

**Keywords:** multiple sclerosis, motor rehabilitation, virtual reality, natural interfaces, augmented reality

## Abstract

**Background:**

The methods used for the motor rehabilitation of patients with neurological disorders include a number of different rehabilitation exercises. For patients who have been diagnosed with multiple sclerosis (MS), the performance of motor rehabilitation exercises is essential. Nevertheless, this rehabilitation may be tedious, negatively influencing patients’ motivation and adherence to treatment.

**Objective:**

We present RemoviEM, a system based on Kinect that uses virtual reality (VR) and natural user interfaces (NUI) to offer patients with MS an intuitive and motivating way to perform several motor rehabilitation exercises. It offers therapists a new motor rehabilitation tool for the rehabilitation process, providing feedback on the patient’s progress. Moreover, it is a low-cost system, a feature that can facilitate its integration in clinical rehabilitation centers.

**Methods:**

A randomized and controlled single blinded study was carried out to assess the influence of a Kinect-based virtual rehabilitation system on the balance rehabilitation of patients with MS. This study describes RemoviEM and evaluates its effectiveness compared to standard rehabilitation. To achieve this objective, a clinical trial was carried out. Eleven patients from a MS association participated in the clinical trial. The mean age was 44.82 (SD 10.44) and the mean time from diagnosis (years) was 9.77 (SD 10.40). Clinical effectiveness was evaluated using clinical balance scales.

**Results:**

Significant group-by-time interaction was detected in the scores of the Berg Balance Scale (*P*=.011) and the Anterior Reach Test in standing position (*P*=.011). Post-hoc analysis showed greater improvement in the experimental group for these variables than in the control group for these variables. The Suitability Evaluation Questionnaire (SEQ) showed good results in usability, acceptance, security, and safety for the evaluated system.

**Conclusions:**

The results obtained suggest that RemoviEM represents a motivational and effective alternative to traditional motor rehabilitation for MS patients. These results have encouraged us to improve the system with new exercises, which are currently being developed.

## Introduction

Multiple sclerosis (MS) is an inflammatory disease in which the insulating cover of nerve cells in the brain and spinal cord are damaged. At present, there are approximately 3 million people who are affected with MS in the world, with rates varying widely in different regions and among different populations. Normally, the disease begins between the ages of 20 and 50, and it is twice as common in women as in men. There is no known cure for MS, but there are several therapies focused to improve function after an attack, preventing new attacks, and preventing disability. Therapies including medication and neurorehabilitation improve some symptoms, but do not change the course of the disease.

The methods used for the motor rehabilitation of patients with neurological problems require the performance of several rehabilitation exercises. These methods have two fundamental problems. First, they propose exercising motor skills by performing the exercises in an insistent and repetitive way. This is not very motivating and decreases the interest of patients to perform them, affecting their adherence to the treatment. Second, these methods require the patients to be in specific centers with the supervision of qualified personnel to ensure correct performance.

The use of new technologies, like virtual reality (VR) and natural user interfaces (NUI), in motor rehabilitation of patients with neurological disorders is well documented [1]. The advantages of VR compared to the traditional methods used for this purpose are: (1) it can recreate different rehabilitation exercises to be performed by the patient in a virtual way (virtual rehabilitation exercises); (2) it can configure the features of rehabilitation exercises, control their performance, and obtain relevant data from the patient performing the exercises; and (3) it can facilitate the interaction between the patient and the system by means of a wide variety of devices.

Furthermore, several studies have demonstrated that by offering virtual rehabilitation exercises as games, greater efficiency is obtained in the rehabilitation process, and the patients are motivated to perform the rehabilitation exercises and their adherence to the treatment is also greater [2-4]. The advantages of using NUI for game consoles with virtual rehabilitation systems are also well documented [5-10].

Some studies focus on the use of technical solutions for the rehabilitation of patients with MS. For instance, the use of VR and augmented reality (AR) for the rehabilitation of the gait [11], or the use of NUI for the rehabilitation of patients with reduced motor skills [12,13]. Nevertheless, this approach of using VR and NUI for motor rehabilitation of MS patients must be more thoroughly explored.

In the case of patients with MS, the performance of motor rehabilitation exercises in the rehabilitation process is even more important, particularly if patients still have good motor skills. MS patients could begin a rehabilitation process by performing virtual rehabilitation exercises using games and interacting with them in an easy and intuitive way, (ie, using NUI that are similar to the ones in game consoles). In this article, we present a Kinect-based virtual rehabilitation system (RemoviEM) that includes all of these features in one motor rehabilitation process. The specific features of the RemoviEM system were previously introduced by the authors in [14]. In this contribution, the authors present a randomized controlled single blinded trial to evaluate the influence of RemoviEM on motor rehabilitation of MS patients. Therefore, the aim of our study is to compare virtual rehabilitation with traditional rehabilitation exercises.

##  Methods

### Trial Registration

Clinical collaborators determined that a public trial registry was not necessary because the features of the system do not create a potential risk for patients. Instead, before patient enrollment, an authorization was given by the clinical center staff. The patients were then informed about the study and all of the participants provided informed consent.

### Participants

There were 56 patients with MS who were potential candidates for this study. The inclusion criteria were: (1) men and women between 18 and 65 years old, (2) patients have relapsing-remitting and secondary-progressive MS, (3) patients have a minimum score of 6 on all items of the domain of the Functional Independence Measure (FIM), (4) patients do not need assistive devices for ambulation or at most a cane, and (5) patients do not have cognitive impairments. The exclusion criteria were: (1) patients with flare-up symptoms, or (2) patients that cannot physically complete all rehabilitation sessions. With these criteria, a final sample of 12 patients was selected and randomly assigned to either the control group (traditional physiotherapy) or the trial group (RemoviEM therapy). All patients were on a similar baseline, with a level and type of impairment constant within groups. The randomization schedule was computer-generated using a basic random number generator. One patient in the control group dropped out of the clinical trial and that data is not included our analysis. Thus, the final sample consisted of 11 patients (7 men and 4 women ranging from 28 to 60 years old). The time since diagnosis for the sample ranged from 2 to 32 years. Age, gender, and chronicity did not affect the performance of each group. The participants had no previous experience with virtual rehabilitation systems. Table 1 shows a summary of the characteristics of the subjects.

**Table 1:** Patient demographics and time since diagnosis.

Issue		Control group, n=5 n (%) or mean (SD)	Trial group, n=6 n (%) or mean (SD)
**Gender, n (%)**			
	Male	4 (80%)	3 (50%)
	Female	1 (20%)	3 (50%)
Age (years)		40.60 (9.24)	48.33 (10.82)
Time since diagnosis (years)		4.70 (3.11)	14.00 (12.69)

### Instrumentation

#### Overview

The software and hardware of RemoviEM will be discussed in detail below. In brief, the program consists of several virtual environments that are designed and developed to allow the patient to perform several motor rehabilitation exercises. The well-developed software allows the therapist to select and configure the exercises to be performed by the patient. The therapist can also follow the patient’s progress in the system. The hardware is composed of low-cost devices that allow virtual environments to be displayed. Interaction with these devices is very intuitive. The low cost of the system facilitates its integration in clinical rehabilitation centers.

#### Software

RemoviEM has three motor rehabilitation exercises: TouchBall, TakeBall, and StepBall.

##### TouchBall

The objective is to work on the balance and weight transfer of the patient, and to perform lateral movements of the trunk. Both issues are essential in order to achieve an improvement in gait and, consequently, to walk correctly.

**Figure 1:**
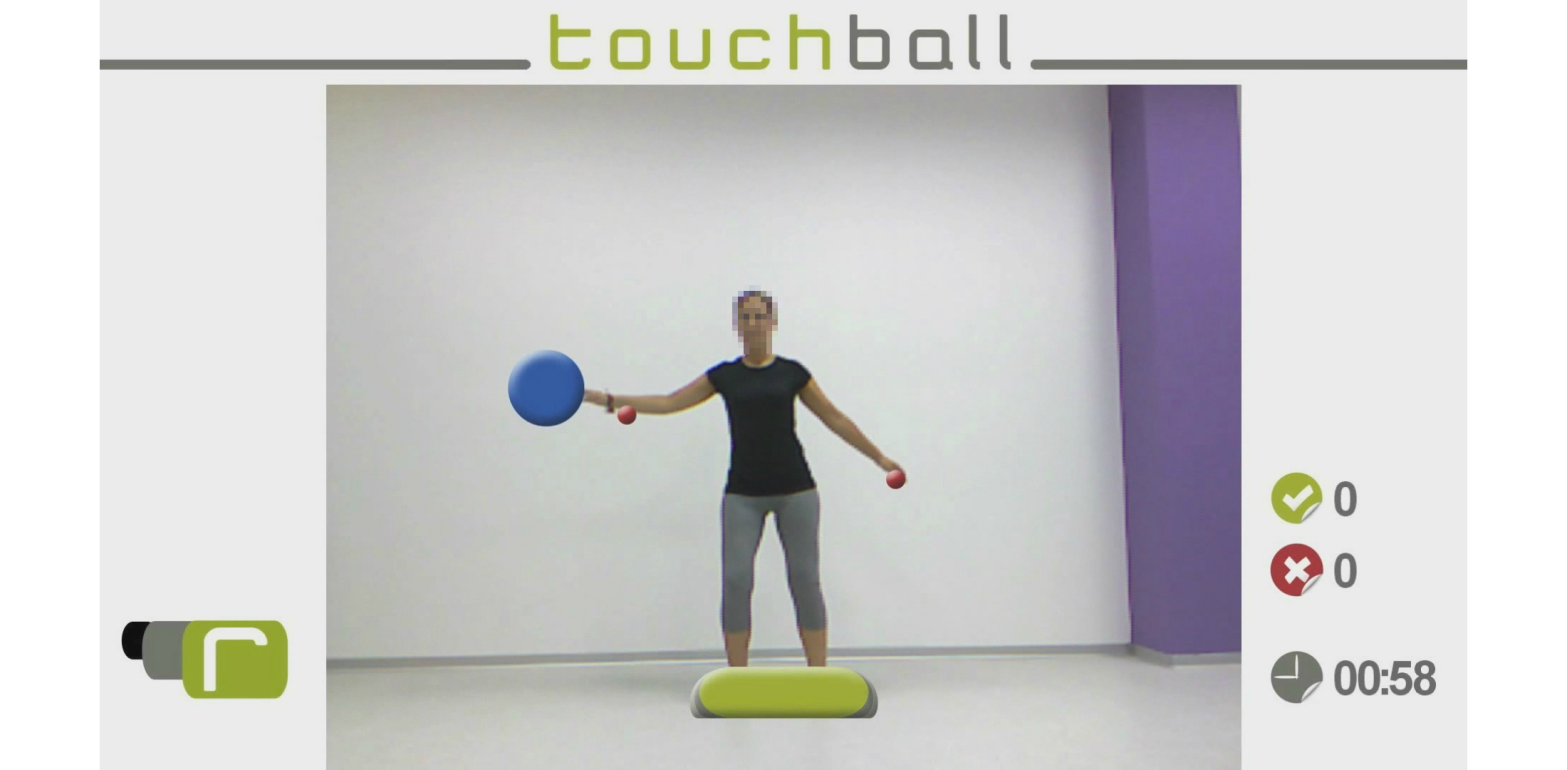
This figure is the TouchBall virtual environment.

In this virtual environment, the patient can interact in a standing or sitting position. Virtual objects appear at different heights on both sides of the patient. The aim of the exercise is to touch virtual objects with your hands, before they disappear, keeping your feet in the predefined zone (Figure 1). The patient has a time limit that is previously established by the therapist to accomplish this. The system also counts the number of hits/misses. If the patient touches the virtual object before it disappears using the hand indicated by the system, and not moving his/her feet outside of the predefined area, the MS patient obtains a hit.

##### TakeBall

The objective is to work the Diagonals of Kabat MMSS. This is very important in neurological rehabilitation because MS patients work on complete movements of the upper limbs, requiring good coordination for their implementation.

**Figure 2:**
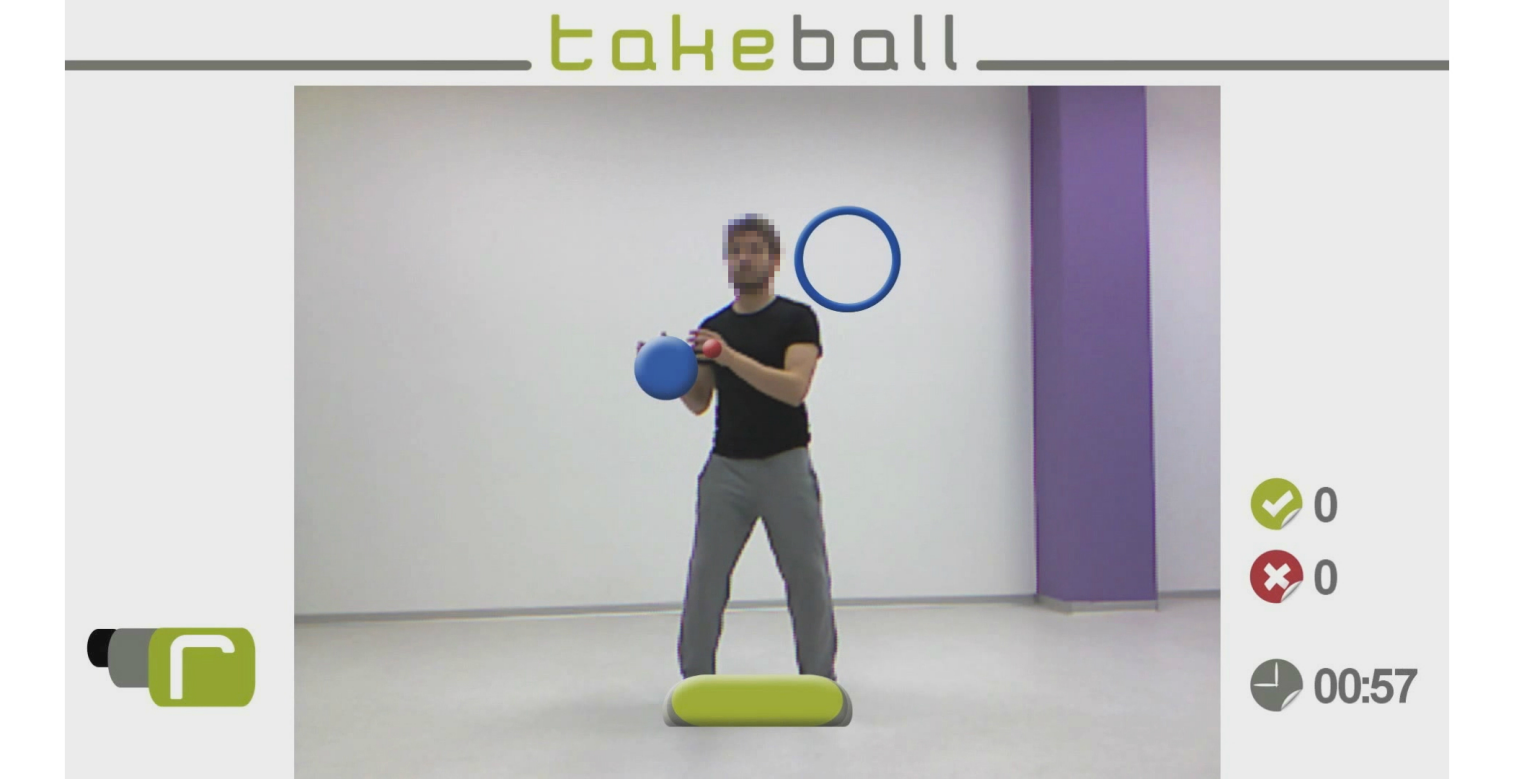
This figure is the TakeBall virtual environment.

In this virtual environment, the patient (in standing or sitting position) must move virtual objects from an initial position to a final position using both hands (Figure 2). The patient has a time limit that is previously established by the therapist to complete the movement. The system also counts the hits and misses. If the patient moves the virtual object from the initial position to the final position before it disappears, the patient obtains a hit.

##### StepBall

The objective is to work balance and weight transfer and to perform lateral movements with monopodal load.

**Figure 3:**
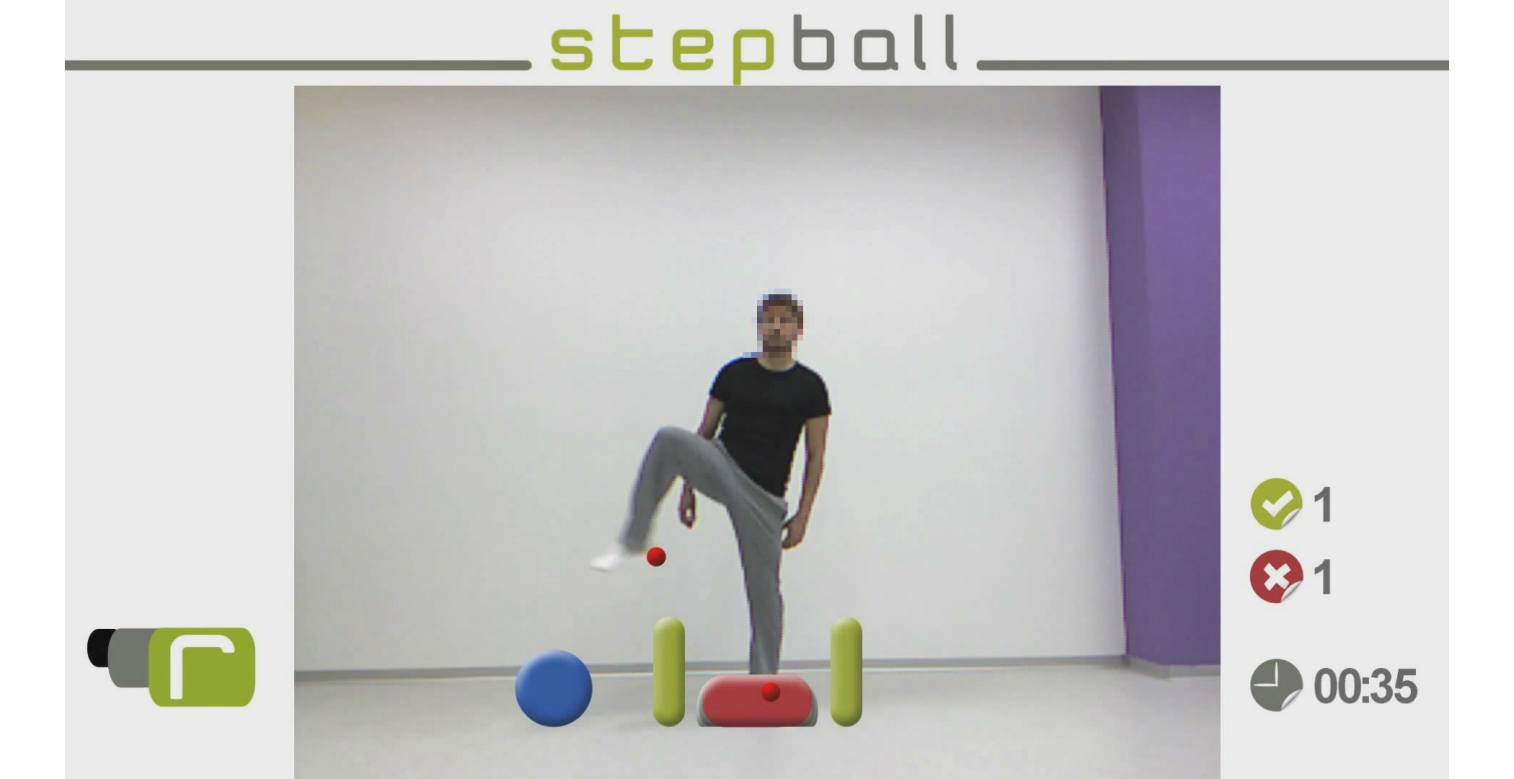
This figure is the StepBall virtual environment.

In this virtual environment, virtual objects appear at ground level on both sides of the patient. The patient must step on the virtual objects before they disappear (Figure 3). To make this movement more difficult, virtual obstacles appear between the original position of the feet and the virtual object. The patient must not touch these obstacles in order to achieve the objective. The patient has a time limit that is previously established by the therapist to step on the virtual object. The virtual environment also counts the number of hits/misses. If the patient steps on the virtual object before it disappears and does not touch the virtual obstacle, the patient obtains a hit.

#### Hardware

RemoviEM uses a LCD/LED TV (42"-47") to show the visual and auditory cues of the virtual environments. In order to interact with the virtual environments, the patient uses Microsoft Kinect. The system runs on a conventional PC.

#### Use

An AR setup is used for the system. The system shows the real image provided by the kinect, where the patient is included. Virtual objects are displayed over this image, allowing the patient to play with the system. The system identifies the different parts of the patient using the depth camera of the kinect. This type of AR allows increases the sense of immersion of the patient to perform exercises.

#### Workflow

The workflow is the same for all virtual environments. Initially, there is a screen that allows the therapist to setup the rehabilitation exercise taking into account the needs and features of the patient. The patient then watches a sequence of explanatory images about interaction with the virtual environment. The goal of these images is for the patient to learn how to interact with the system to be able to perform the rehabilitation exercise. Next, there is a new screen in which the patient can see how the system detects the skeleton of the patient’s body, and also whether or not the patient is correctly positioned to perform the rehabilitation exercise. When the system detects that the body is properly positioned, a countdown allows the patient to prepare for the start of the exercise. Finally, at the end of the rehabilitation exercise, the system shows the patient and the therapist a final screen with a summary of the results obtained.

### Intervention

The clinical trial was carried out in the neurorehabilitation service of the Multiple Sclerosis Association of Castellon (Asociación de Esclerosis Múltiple de Castellón, AEMC). Each patient participated in 10 one-hour sessions of rehabilitation and completed one session per week.

In each session, the patients in the control group (n=5) performed standard balance and gait rehabilitation exercises. The patients belonging to the experimental group (n=6) spent forty-five minutes performing the same exercises, and during the last fifteen minutes of the session, they performed the virtual rehabilitation exercises.

Before and after the rehabilitation program, all the patients were assessed by a specialist. This clinical assessment used several scales. Balance in static condition was assessed by the Berg Balance Scale (BBS) [15], the Tinetti Balance Scale [16], and the Single Leg Balance test (SLB). Balance in dynamic conditions was assessed by the 10-meter Walking Test (10MT) [17], and the Time “Up and Go” Test (TUG) [18,19]. In order to obtain subjective information about the treatment, a feedback questionnaire (the Suitability Evaluation Questionnaire, SEQ; [20]) was given to th patients.

### Data Analysis

Repeated measure analyses of variance (ANOVAs) with time (before and after rehabilitation), the within-subjects factor and treatment option (control versus trial), and the between-subjects factor were performed for each one of the balance measures. The main effects of time, treatment option, and the time-treatment option interaction effects were evaluated. Simple contrasts were conducted for each significant time main effect to determine the source of the significant difference. A *P* value less than .05 was considered to be significant in each case.

## Results

No significant differences in demographical (age and gender) or clinical (chronicity) variables at inclusion were detected between groups (*P*=.240, *P*=.353 and *P*=.147 for age, gender, and chronicity, respectively).

A repeated measure ANOVA at the beginning and at the end of the clinical trial revealed a significant time effect for the BBS (*P*=.014), Tinetti (*P*=.003), SLB test right foot (*P*=.041), and 10MW (*P*<.001). Nevertheless, there were no significant time effects for SLB left foot (*P*=.052) or TUG (*P*=.346). No group effect was detected for any outcome, except for TUG (*P*=0.027). Finally, significant group-by-time interaction was detected in the scores for BBS (*P*=.030) and SLB test right foot (*P*=.033) (Table 2). In addition, all the patients said they had fun during the treatment, and they did not suffer any discomfort (disorientation, cyber-sickness or adverse symptoms) when performing the virtual rehabilitation exercises.

The score of the SEQ for Virtual Rehabilitation systems [20] was 55.560/65 (SD 5.940). In addition, all the patients remarked that they had fun during the treatment. There was only one reported case of a patient not being in control of the exercises. None of the patients suffered from spatial disorientation or cyber-sickness, and no adverse symptoms were described by the therapists.

**Table 2:** Numerical data of the scores of scales and tests in the assessments carried out before and after the treatment. G: group effect; T: time effect; GxT: group/time effect.

Test		Before treatment		After treatment		Difference	Significance		
		Mean	SD	Mean	SD	Mean	GxT	G	T
**BBS**							0.030	0.546	0.014
	Control	51.400	6.309	51.600	5.899	0.200			
	Trial	48.000	6.325	50.333	5.630	2.333			
**Tinetti**							0.716	0.412	0.003
	Control	25.000	2.236	26.000	2.449	1.000			
	Trial	23.830	2.563	24.670	2.422	0.840			
**SLB left foot**							0.463	0.685	0.052
	Control	15.378	14.172	18.672	15.540	3.294			
	Trial	10.721	8.105	17.440	10.680	6.719			
**SLB right foot**							0.033	0.650	0.041
	Control	18.692	15.509	18.517	15.743	-0.175			
	Trial	11.730	9.179	18.339	10.604	6.609			
**TUG**							0.652	0.027	0.346
	Control	8.590	4.608	6.930	1.812	-1.660			
	Trial	10.692	3.468	8.318	2.063	-2.374			
**10MW**							0.472	0.531	0.000
	Control	16.625	6.064	14.490	4.597	-2.135			
	Trial	19.161	6.299	16.469	5.770	-2.693			

## Discussion

### Principal Findings

The study presented in this paper assessed the influence of a KINECT-based virtual rehabilitation system (RemoviEM) on the rehabilitation of patients with MS.

In relation with the static and dynamic balance abilities of the patients, measured with the Berg Balance Scale, there was a significant improvement over time on this scale. While the control group remained virtually stagnant over time, the experimental group showed a clear improvement. The results provide evidence of significant improvement in the experimental group compared to the control group over time.

Regarding the Tinetti Balance Scale, other common clinical test used to assess the static and dynamic balance abilities of the patients, both groups showed a similar trend. There was a significant improvement over time, although there were no significant differences over time between the two groups. The Tinetti Balance Scale measures balance as well as gait, but our system is focused only in balance; this partially explains why there were no significant differences between the groups overtime.

The Single Leg Balance test shows that the improvement in the experimental group was greater than the control group for both the right and left feet. This was statistically significant in the right foot (**P*=.*033).

In the Time “Up and Go” test, used to assess a person's mobility, the improvement in the experimental group was higher than in the control group; however this improvement was not statistically significant. RemoviEM does not include specific training exercises “Sit-To-Stand”, and the inclusion of such exercises would allow Time “Up and Go” test results to be better since they would train one of the basic parts evaluated by this test.

In relation to the 10-meter Walking Test, used to assess walking ability of patients, the results showed improvement over time, but no improvement was detected in significant group-by-time, which suggests that both groups improved similarly. This was likely due to the fact that the RemoviEM system does not specifically train gait.

The specialist also said that the patients were interested in performing virtual exercises with RemoviEM, and that the patients were entertained to a greater extent than when doing traditional exercises. All of this was found without detecting any cyber side effect in the MS patients. Thus, the RemoviEM system obtained additional motivation and adhesion to the treatment by the patients.

### Limitations

The results obtained in most of the scales used show a significant improvement in the experimental group compared to the control group. From this perspective, we can confirm that virtual rehabilitation with RemoviEM is capable of improving the rehabilitation of MS patients. Nevertheless, we know that the small size of the sample should be taken into account, when analyzing these results. The deviation of several test is too high, thus the qualitative information of the associated means is reduced. A new study enrolling more patients could solve this problem.

Basically, RemoviEM includes rehabilitation exercises to work movements of the upper limbs, to acquire good coordination, to improve the balance and weight transfer of the patient, and to perform lateral movements of the trunk, with or without monopodal load. The improvements obtained in the present study are specifically in BBS, weight transfer, and TUG (ie, in the scales measuring the aspects mentioned above).

Nevertheless, more rehabilitation exercises to train specific movements like Sit-To-Stand, and other generic movements like gait and running should be incorporated in RemoviEM. Thus, it would be possible to study whether these improvements would also be significant on Tinetti and 10MW.

### Conclusions

RemoviEM has helped patients with MS become more motivated and involved in the rehabilitation process. In addition, the exercises included in the system have allowed patients to obtain significant improvements in the rehabilitation of those movements for which they were designed. We think that the inclusion of new exercises that are designed for the treatment of other movements but that are not currently addressed can also give satisfactory results. Currently, a second stage of the RemoviEM system is being designed to include these new rehabilitation exercises, taking into account the suggestions of clinical specialists and MS patients.

## Abbreviations

AR: augmented reality
BBS: Berg Balance Scale
MS: multiple sclerosis
NUI: natural user interfaces
SEQ: suitability evaluation questionnaire
TUG: Time "Up and Go" test
VR: virtual reality
